# Within-Host Diversity of SARS-CoV-2 in COVID-19 Patients With Variable Disease Severities

**DOI:** 10.3389/fcimb.2020.575613

**Published:** 2020-10-06

**Authors:** Hebah A. Al Khatib, Fatiha M. Benslimane, Israa E. Elbashir, Peter V. Coyle, Muna A. Al Maslamani, Abdullatif Al-Khal, Asmaa A. Al Thani, Hadi M. Yassine

**Affiliations:** ^1^Biomedical Research Center, Qatar University, Doha, Qatar; ^2^Virology Laboratory, Hamad Medical Corporation, Doha, Qatar; ^3^Communicable Diseases Center, Hamad Medical Corporation, Doha, Qatar

**Keywords:** SARS-CoV-2, COVID-19 severity, nonsynonymous mutations, virus quasispecies, within-host diversity

## Abstract

**Background:** The ongoing pandemic of SARS-COV-2 has already infected more than eight million people worldwide. The majority of COVID-19 patients either are asymptomatic or have mild symptoms. Yet, about 15% of the cases experience severe complications and require intensive care. Factors determining disease severity are not yet fully characterized.

**Aim:** Here, we investigated the within-host virus diversity in COVID-19 patients with different clinical manifestations.

**Methods:** We compared SARS-COV-2 genetic diversity in 19 mild and 27 severe cases. Viral RNA was extracted from nasopharyngeal samples and sequenced using the Illumina MiSeq platform. This was followed by deep-sequencing analyses of SARS-CoV-2 genomes at both consensus and sub-consensus sequence levels.

**Results:** Consensus sequences of all viruses were very similar, showing more than 99.8% sequence identity regardless of the disease severity. However, the sub-consensus analysis revealed significant differences in within-host diversity between mild and severe cases. Patients with severe symptoms exhibited a significantly (*p*-value 0.001) higher number of variants in coding and non-coding regions compared to mild cases. Analysis also revealed higher prevalence of some variants among severe cases. Most importantly, severe cases exhibited significantly higher within-host diversity (mean = 13) compared to mild cases (mean = 6). Further, higher within-host diversity was observed in patients above the age of 60 compared to the younger age group.

**Conclusion:** These observations provided evidence that within-host diversity might play a role in the development of severe disease outcomes in COVID-19 patients; however, further investigations are required to elucidate this association.

## Introduction

Coronaviruses are a group of single-stranded, positive-sense RNA viruses that infect a wide range of vertebrates (Cui et al., 2019). There are four human coronaviruses that are seasonal viruses and cause mild upper respiratory tract infections: OC43, 229E, HKU1, and NL63 (Weiss and Navas-Martin, 2005). In the last two decades, nevertheless, there have been two major outbreaks of highly pathogenic human coronaviruses that emerged from zoonotic origins (Amanat and Krammer, 2020). The first outbreak occurred in 2003 in China due to the emergence of severe acute respiratory coronavirus (SARS-CoV-1) from civet cats. The outbreak resulted in more than 9,000 infections and 774 deaths in 37 countries (de Wit et al., 2016). The second outbreak occurred in 2012 in Saudi Arabia and was caused by the Middle East respiratory syndrome-related coronavirus (MERS-CoV). The virus continues to circulate in camels and has infected around 2,500 people and resulted in 858 deaths after 8 years of its emergence (de Wit et al., 2016). In December 2019, a third highly pathogenic coronavirus, SARS-CoV-2, has spread among humans resulting in the ongoing pandemic and massive socioeconomic losses around the globe. As of May 2020, the virus has infected more than eight million people and resulted in more than 380,000 deaths (World Health Organization, 2020). Generally, more than 85% of patients either are asymptomatic or show mild manifestations such as fever, cough, and fatigue. However, around 15% of patients suffer from life-threatening complications that can lead to death (Chen et al., 2020). Severe complications might include the development of acute respiratory distress syndrome (ARDS), severe pneumonia, and chronic lung damage (Wolfel et al., 2020; Xu et al., 2020). Several questions remain unanswered regarding the determinants of disease severity. Current knowledge attributes variabilities in disease outcomes to host-related factors such as age and chronic illness, and infection-related factors such as lymphocytopenia and cytokine storm, rather than variations in viral genetics (Zhang et al., 2020). Previous studies have shown that the within-host genetic diversity of RNA viruses may impact virus virulence (Vignuzzi et al., 2006), immune escape (Nowak et al., 1991), and drug resistance (Johnson et al., 2008). Further, within-virus diversity has been found to contribute to disease severity in some medically important RNA viruses such as hepatitis C virus (HCV) and human immunodeficiency virus (Sullivan et al., 2007; Lauring and Andino, 2010; Wu et al., 2017). The within-host diversity was also reported in infected patients during SARS-CoV-1 and MERS coronaviruses outbreaks; however, little is known about their effect on virus evolution, transmissibility, and pathogenies (Xu et al., 2004; Lauring and Andino, 2010; Briese et al., 2014; Park et al., 2016). To date, thousands of SARS-CoV-2 sequences have been deposited in public databases; yet, we still lack fundamental information about the within-host diversity of SARS-CoV-2 and its possible role in disease severity. Here, we compared the SARS-CoV-2 diversity in COVID-19 patients with mild and severe manifestations and investigate its impact on clinical disease severity.

## Methods

### Study Groups and Sample Collection

Nasopharyngeal swabs were collected from COVID-19 patients with variable disease outcomes during the period from Mar 13, and Apr 10, 2020. A total of 46 samples were collected from 19 mild cases and 27 severe cases. Basic demographic and clinical information of patients is listed in Table 1. Patients with mild clinical manifestations showed symptoms such as fever, cough, and malaise. Patients with severe symptoms were categorized based on the Sofa score system into five categories (Cardenas-Turanzas et al., 2012). Associated comorbidities were reported in 23 patients. Most common reported comorbidities were hypertension (65%), diabetes (60%), cardiovascular diseases (26%), and asthma (13%). This study was approved by IRB committees of Hamad Medical Corporation (MRC-01-20-145) and Qatar University (QU-IRB 1289-EA/20).

**Table 1:** Summary of the main demographic and clinical characteristics of COVID-19 patients included in this study.

	**Samples**	**Gender**		**Median age (age range)**	**Comorbidities**				Severity score^*^ (only severe cases)				
		**F**	**M**		**0**	**1**	**2**	**>3**	**0**	**1**	**2**	**3**	**4**
Mild cases	19	3	16	34 (20–54)	14	4	1	0	—				
Severe cases	27	6	21	51 (19–72)	9	6	5	7	4	3	6	8	6

* *Severity score was calculated using Sofa score (Cardenas-Turanzas et al., 2012)*.

### Virus Genome Sequencing and Data Analysis

Viral RNA was extracted directly from viral transport media using QIAamp Viral RNA Mini kit (Germany, Qiagen), according to the manufacturer's instructions. A total of 100 ng of extracted RNA was reverse transcribed with N8 random hexamers. Second-strand synthesis, PCR amplification, and full virus genome sequencing were done using CleanPlex SARS-CoV-2 Research and Surveillance Panels provided by Paragon Genomics (Shenzhen, China). Indexed libraries were purified with Agencourt AMPure XP beads (Beckman Coulter, USA), quantified using Qubit 4.0 Fluorometer (Invitrogen, USA), and pooled to a final concentration of 8 pM. Pooled libraries were then loaded in a 300-cycle sequencing cartridge. Sequencing was performed on an Illumina MiSeq platform, which generated about six GB of data. The mean count of paired sequencing reads per sample was 1.5 × 10^6^ (minimum 0.5 × 10^6^ to maximum 3.5 × 10^6^).

The generated sequencing reads were first trimmed to remove adaptors using the cutadapt software (https://cutadapt.readthedocs.io/en/stable/). Additional filtration steps also included the removal of short reads (<40 nucleotides) and low-quality reads (<30 Phred score). High-quality sequencing reads were then aligned to the reference genome (accession number: MN908947) using Burrows-Wheeler Alignment (BWA) (Li and Durbin, 2009). Consensus sequences were constructed after trimming the PCR primer sequences using fgbio software package (https://github.com/fulcrumgenomics/fgbio). We were able to generate near-full genomes (from nucleotide 10 to nucleotide 29,700) from all samples with an average coverage of 98%. For variant calling, gatk tools were used for variant detection at both consensus and sub-consensus levels (https://gatk.broadinstitute.org). Only variants with Phred scores of more than 35 and depths of more than 100× were called. For low-frequency variant calling (frequency >10%), reads were realigned to consensus sequences reconstructed from each sample. A phylogenetic tree was generated employing the general-time reversible (GTR+G) nucleotide substitution model with 1,000 bootstrap replicates and was displayed using FigTree (version 1.4.4) (Yang, 1994). Lineages were assigned to sequences based on the lineage nomenclature system proposed by Rambaut et al. (2020). Statistical analysis was performed using Prism7 software.

## Results

### Analysis of SARS-CoV-2 Nucleotide Variation

**Figure 1:**
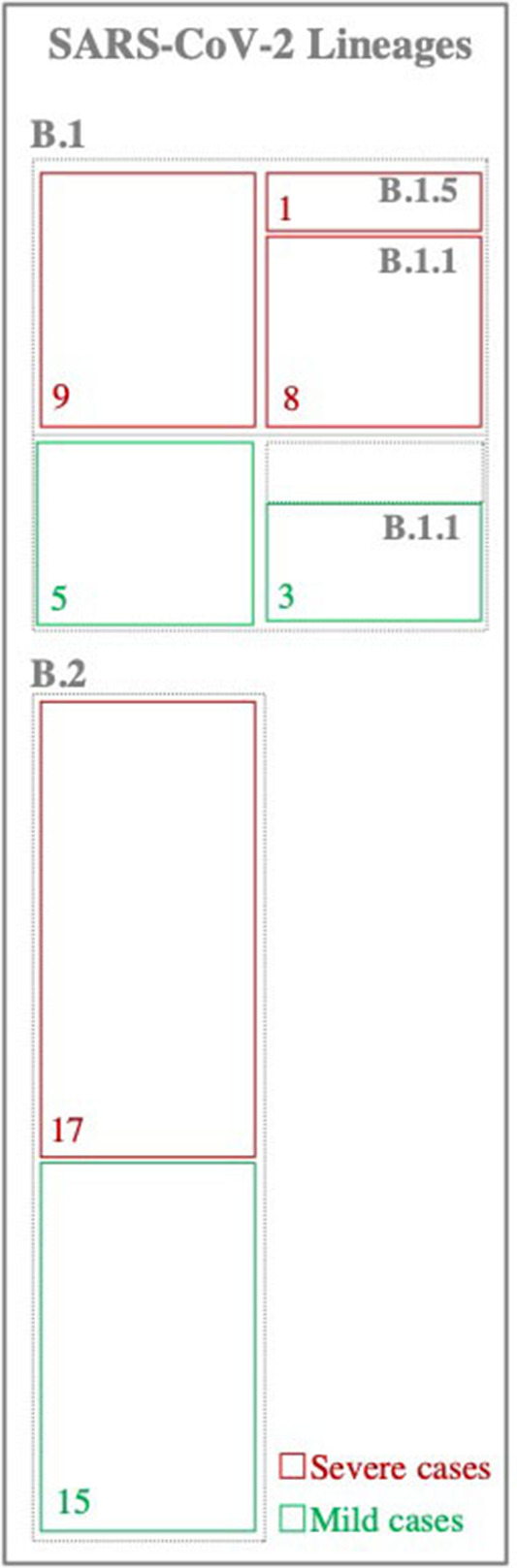
Classification of SARS-CoV-2 viruses. Viruses were classified based on the lineage nomenclature system proposed in Rambaut et al. (2020). Numbers inside the boxes represent the total number of samples within each lineage.

Phylogenetic analysis of viruses consensus sequences revealed the clustering of SARS-CoV-2 viruses into two major lineages: B.1 (*n* = 14 viruses) and B.2 (*n* = 32 viruses) lineages (Figure 1). Of note, there were no differences in the clustering patterns of viruses from mild and severe cases.

**Figure 2:**
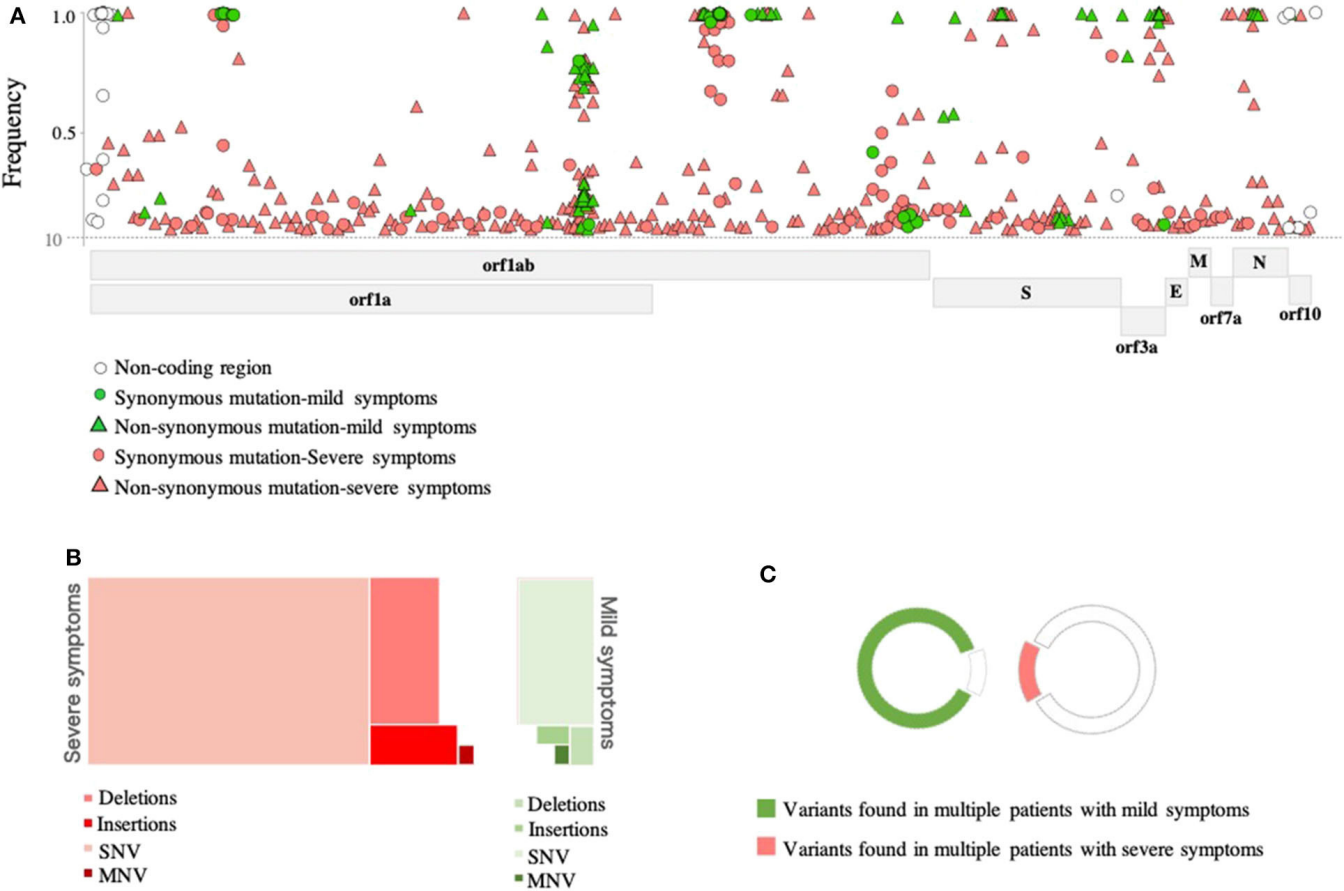
Genomic variations detected in SARS-CoV-2 viruses. **(A)** Genomic positions and frequencies of variants found in the SARS-CoV-2 genome (55-29640) of 46 patients. The y-axis represents the frequency of variants, and the x-axis represents the genomic position of variants. **(B)** A tree map presenting types of variants found in SARS-CoV-2 genomes of mild (green) and severe (red) cases. **(C)** Colored parts of the circles represent variants seen in more than one patient.

In-depth sequence analysis revealed a total number of 236 variants with frequency >10% in all SARS-CoV-2 genomes (*n* = 46 samples) with respect to the reference sequence (MN908947). Overall, the majority (80%) of variants detected in severe cases were found at the sub-consensus level (frequency <50%). In contrast, 40% of variants were found at the sub-consensus level in mild cases (Figure 2A). In both cases, variants were mainly located in ORF1ab (*n* = 164; 69%), while 34 (14%) variants were detected in the spike (S) gene. A limited number of variants were reported in other coding regions, including nine variants in ORF3a, three in the envelope (E) gene, four variants in the matrix (M) gene, 11 variants in the nucleocapsid (N) gene, and three in ORF10. Additional nine variants were also found in non-coding regions of the genome, mainly at the 5′- and 3′- end of the genome. A total of 59 synonymous and 177 non-synonymous variants were identified in these eight protein-coding regions (Figure 2A). Notably, the majority of non-synonymous variants were detected at the sub-consensus level (frequency <50%) and never reached consensus sequences. This may suggest that purifying selection eliminates non-synonymous mutations before they reach the consensus level. Further, most of the identified non-synonymous variants (152 out of 177) were detected exclusively in severe cases compared to only 10 unique non-synonymous variants in mild cases. Similarly, 49 (out of 59) of identified synonymous variants were found exclusively in severe cases compared to only five specific synonymous variants in mild cases. Deletions and insertions (indels) represented 18 and 6% of reported non-synonymous variants, respectively, in severe cases. Comparably, indels accounted for 10 and 8% of variations in mild cases. Only one multiple nucleotide variant (MNV; GGG28881-AAC28883) was detected in the N gene of both groups of patients but with different prevalence (15% in mild cases vs. 41% in severe cases) (Figure 2B). Further analysis of sequences at the sub-consensus level showed that the majority (84%) of variants found in patients with severe symptoms were rarely seen in more than one patient, while more than 85% of variants in mild cases were found in multiple patients (Figure 2C).

**Figure 3:**
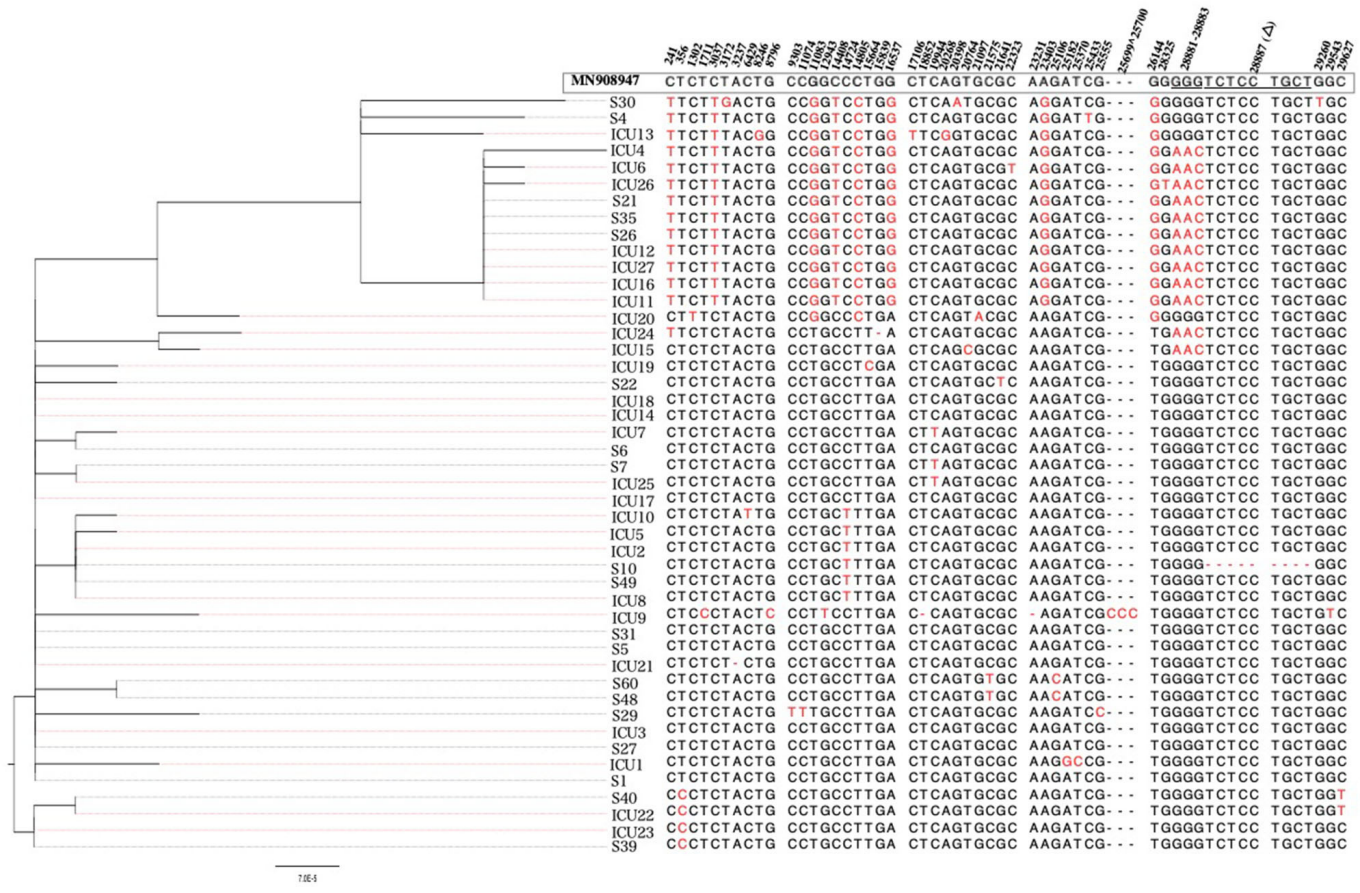
Phylogenetic tree of sequenced SARS-CoV-2 viruses (*n* = 46 samples). Maximum likelihood phylogeny was reconstructed under the general-time reversible model (GTR) as inferred by model testing. Nucleotide sequence alignment shows mutations found at the consensus level (>50% frequency) with respect to the reference strain (MN908947).

At the consensus level (frequency >50%), all sequences were showing an overall similarity of >99.9% to Wuhan-Hu-1 strain regardless of disease severity (Figure 3). Only 54 variants (out of 236) were found at the consensus level. Of these, 14 variants were found in both mild and severe cases. Again here, viruses from severe cases were carrying twice the number of variants (*n* = 42 variants) compared to viruses from mild cases (*n* = 23 variants). Also, only 11 out of 47 indels appeared in the consensus sequences of some viruses. Eight of which were seen in viruses from severe cases and three in viruses from mild cases. In both groups of patients, most of the reported indels were located in the ORF1ab and were rarely seen in more than one patient.

**Figure 4:**
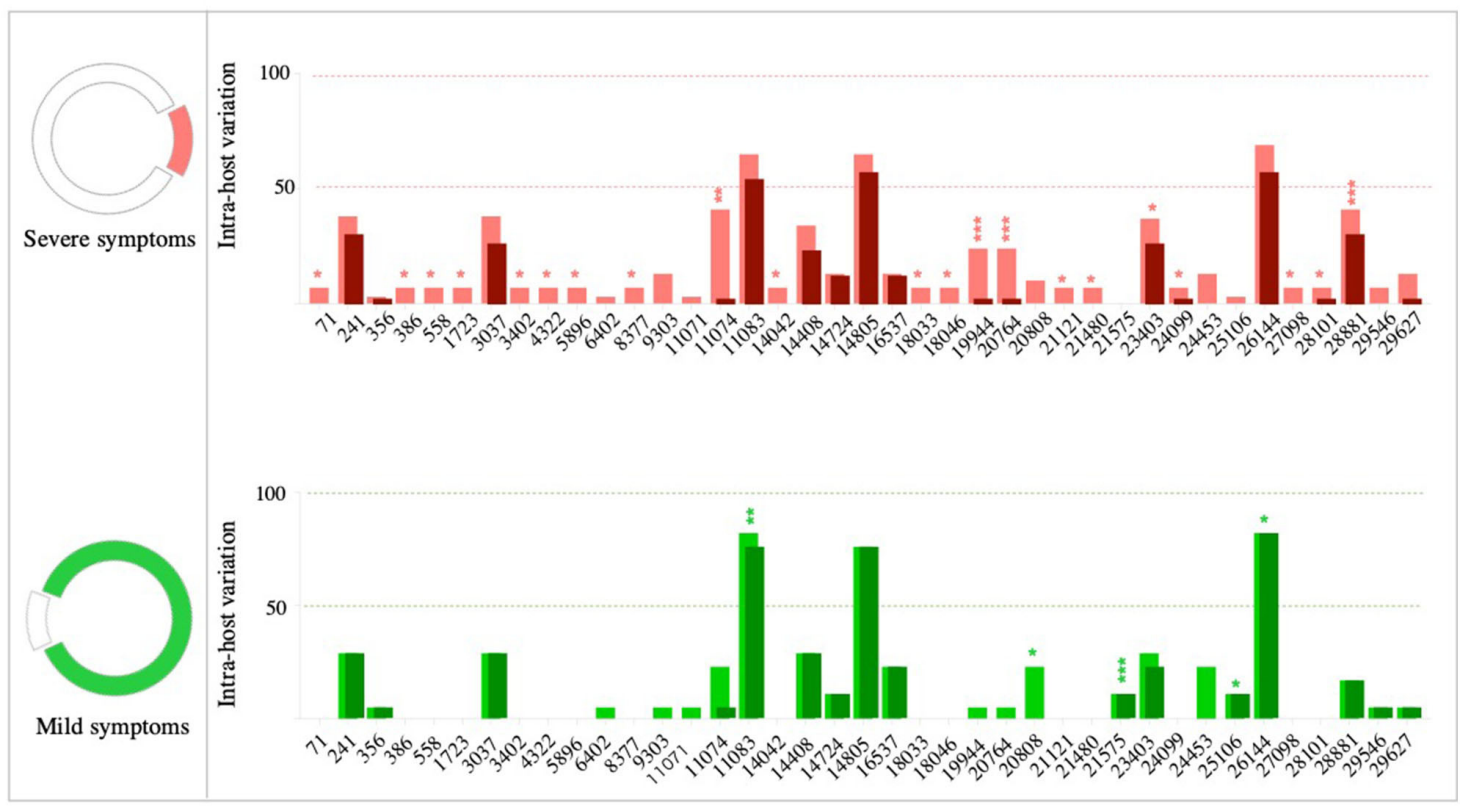
Comparison of variant prevalence in mild cases (green) and severe cases (red). Colored parts of the circles (on the left) represent variants seen in more than one patient. Only variants found in more than one patient were used to generate bar charts. Bar charts demonstrate the prevalence of each variant in patients within each group (severe vs. mild). Bars with darker colors demonstrate variants detected at the consensus sequence level. *P*-values are indicated as follows: ****p < 0.0001, ***p < 0.001, **p < 0.01, and *p < 0.05.

Then, we compared the presence and prevalence of variants—at both consensus and sub-consensus levels—in patients with severe and mild symptoms. To do so, we only included variants found in more than one patient (*n* = 39 variants). Notably, 16 of these variants were detected exclusively in severe cases; however, none of these variants was seen in more two patients (Figure 4). In contrast, the non-synonymous variant, 21,575, was the only variant exclusively found in mild cases but also at a relatively low prevalence (11%; *p*-value 0.0007). Variants at positions 19,944 (synonymous; ORF1ab), 11,074 (insertion; ORF1ab), 20,764 (synonymous; ORF1ab), and 28,881–28,883 (MNV; N) were found in both groups but at significantly higher prevalence (*p* < 0.009) among severe cases. Despite the significant differences between the two groups at the sub-consensus level, a mutation pattern was more similar at the consensus level. Analyzing variants at the consensus level revealed only three differentially prevalent, all non-synonymous, variants between mild and severe cases. Those variants included two S variants: 21,575 and 25,106, which were found at significantly higher prevalence (*p*-value 0.0007 and 0.049, respectively) in mild cases, and one N variant: 28,881–28,883, which was significantly higher among severe cases (*p*-value 0.0003) (Figure 4). Further investigation on the effect of these variants is important to explain these differences between patients with variable clinical outcomes.

**Figure 5:**
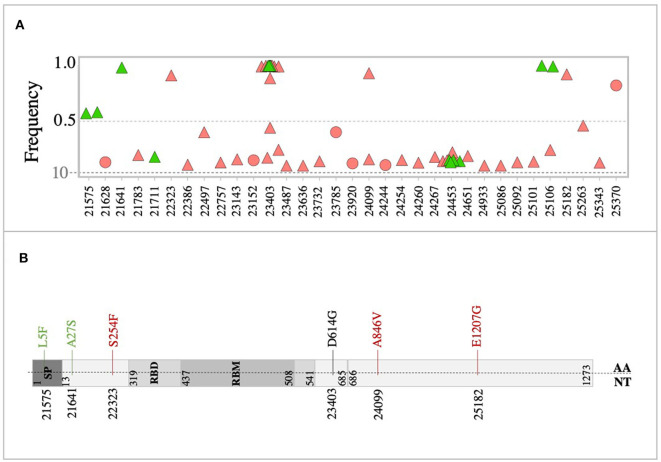
Variants in the SARS-CoV-2 spike glycoprotein. **(A)** Genomic position and frequency of variants detected in S genes of all viruses. **(B)** Schematic representation showing consensus mutations found in mild (green) and severe (red) cases. D614G mutation was found in both mild and severe cases.

Analysis of the spike (S) glycoprotein revealed 31 variants in the S genes of all sequenced viruses at both consensus and sub-consensus levels. Overall, the majority of S variants were non-synonymous variants (*n* = 25) and were mainly detected at the sub-consensus level (Figure 5A). Also, the majority of S variants (*n* = 24 variants) were exclusively detected in severe cases, compared to only three unique variants in mild cases (Figure 5A). All reported S variants were sporadically detected, except for four variants: 23,403 (29% in mild and 48% in severe), 24,099 (6% in severe), 24,453 (23% in mild and 13% in severe), and 25,106 (11% in mild and 3% in severe). Interestingly, only one of the sub-consensus variants, 22,757, corresponding to S399P, was found in the receptor-binding domain (RBD) of S protein. It was detected once among severe cases.

At the consensus level, only eight variants (out of 31) were found in all consensus sequences (Figure 5B). Of these, variants 21,575 (11%) and 21,641 (5%) were found in viruses from mild cases, while variants 22,323 (3%), 24,099 (7%), and 25,182 (3%) were found in severe cases only. The two groups of patients shared only one non-synonymous variant, 23,403, corresponding to amino acid change D614G (Figure 5B). This mutation, in particular, was seen at the consensus level of 29% mild cases, while it was seen at both consensus and sub-consensus sequences of 38 and 10% of severe cases, respectively. None of the consensus mutations was found in the receptor-binding domain (RBD), which might indicate adaptation to human receptors.

### Analysis of Within-Host Diversity of SARS-CoV-2

**Figure 6:**
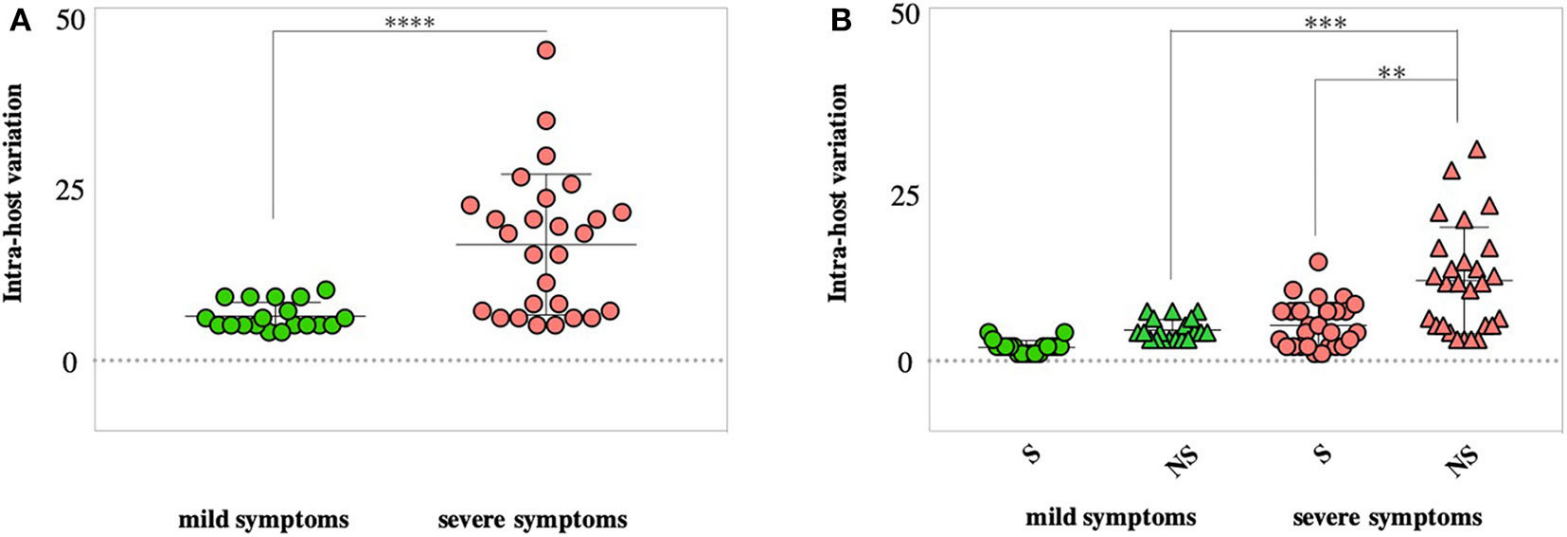
Assessment of within-host SARS-CoV-2 diversity. **(A)** Total number of variants detected in each patient with mild (green) and severe (red) symptoms. **(B)** Number of synonymous (S) and non-synonymous (NS) mutations found within each patient of the two groups (mild vs. severe cases). *P*-values are indicated as follows: ****p < 0.0001, ***p < 0.001.

We then compared within-host diversity between mild and severe COVID-19 cases by assessing the total number of variants (frequency >10%) detected within each patient. Overall, the average number of within-host variants ranged from 4 to 10 variants (mean = 6.2; SD 1.9) in mild cases. On the other hand, the number of within-host variants was significantly higher (*p* < 0.0001) in severe cases, ranging from 5 variants to 44 variants (mean = 16.4; SD 9.9) (Figure 6A). In-depth analysis of within-host variants revealed the dominance of non-synonymous variants (mean = 11.3; SD 3.2) over synonymous variants (mean = 5; SD 7.5) in patients with severe symptoms (*p* < 0.0001). In contrast, no significant difference was reported in the number of non-synonymous (mean = 4.3; SD 0.9) and synonymous (mean = 1.9; SD 1.4) variants in mild cases (Figure 6B). Additionally, the within-host diversity was more consistent among mild cases compared to severe cases who showed higher variabilities (Figures 6A,B). A subset of severe cases exhibited within-host diversity patterns similar to those with mild symptoms; all were patients under the age of 60. Within-host diversity in mild cases was mainly comprised of variants found at the consensus level (frequency >50%). On average, mild cases were exhibiting two sub-consensus variants, ranging from 0 to 5 variants per patient. In contrast, the higher within-host diversity in severe cases was mainly due to variants found at the sub-consensus level. On average, severe cases exhibited 10 sub-consensus variants, ranging from 0 to 28 variants per patient. Taken together, these results suggest that SARS-CoV-2 exists as a complex and dynamic distribution of variants within infected patients, rather than a single genomic sequence, in particular, among patients with severe symptoms.

**Figure 7:**
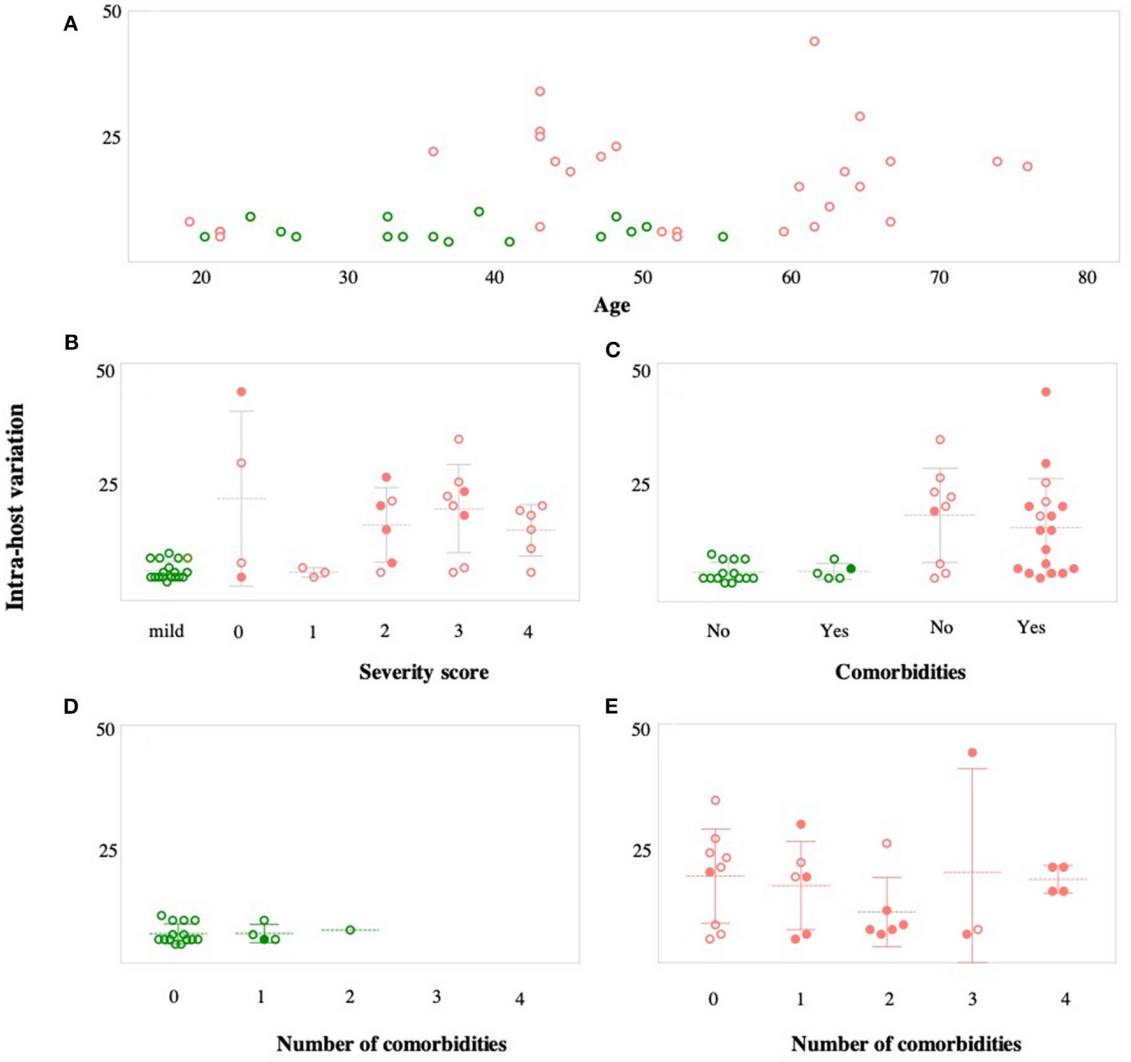
Correlation between within-host variation (number of variants within each patient) and age **(A)**, severity score **(B)**, and number of comorbidities **(C)**. Patients with mild symptoms are indicated in green while patients with severe symptoms are indicated in red. Shaded circles in **(B–E)** indicate patients older than 50 years.

In the next part of within-host diversity analysis, we investigated the association between within-host diversity and (i) patient age, (ii) disease severity, and (iii) number of associated comorbidities (Figure 7). There was a significant relationship between within-host diversity and age (Wilcoxon *p*-value 0.001) (Figure 7A). As expected, more than half of patients (55%) with severe symptoms were above the age of 50 and 34% were above the age of 60. None of the patients with mild symptoms, on the other hand, was above the age of 60. Higher within-host diversity was reported among severe cases above the age of 60. Additionally, within-host diversity was also high among severe cases who were in their 40 s (*n* = 7 patients) (Figure 7A). Those seven patients had severity scores of 2 (in 2 cases), 3 (in 4 cases), and 4 (in one case). Considering the severity levels, results showed an overall higher within-host diversity in severe cases, classified as having a severity score of “3,” compared to patients with severity scores of “2” and “1.” However, the average within-host diversity was less among patients with a severity score of “4” (Figure 7B). Despite the significant differences in within-host diversity between mild and severe cases, these differences were not obviously seen among severe cases with different severity scores (Figure 7B). Finally, studies on COVID-19 patients have documented an increased probability of intensive care admission among patients with comorbidities (Grasselli et al., 2020; Guan et al., 2020a; Vardavas and Nikitara, 2020). Therefore, we compared within-host diversity between patients with and without comorbidities and found no significant differences (Figure 7C). We also assessed the correlation between within-host diversity and the number of associated comorbidities, but again, there was no evidence of a correlation between the two factors (Figures 7D,E). Overall, our observations suggest that the remarkably high within-host diversity seen in severe cases might play a role in disease severity; however, the underlying mechanism is still to be investigated.

## Discussion

Despite the unprecedented efforts to sequence the SARS-CoV-2, the majority of published data report the virus genetic diversity at the consensus level. This, however, was found to be insufficient for understanding the evolution, transmission, and pathogenicity of RNA viruses (Domingo et al., 1985; Holmes and Moya, 2002). Furthermore, viral quasispecies has previously been reported in seasonal coronaviruses such as HKU1 and OC43 (Vabret et al., 2006; Gorse et al., 2017; Woo et al., 2017), as well as zoonotic coronaviruses such as SARS-CoV-1 and MERS (Xu et al., 2004; Tang et al., 2006; Borucki et al., 2016). These findings suggest the existence of coronaviruses as quasispecies within infected patients, similar to other RNA viruses. Besides, within-host diversity of some coronaviruses such as OC43 was found to be associated with acute respiratory illness in infected patients (Vabret et al., 2006). The correlation between virus quasispecies and disease pathogenesis has been thoroughly investigated in HCV and HIV viruses (Farci et al., 2000; Cabot et al., 2001; Qin et al., 2005; Dampier et al., 2016). Recently, similar studies have been conducted in other RNA viruses, such as influenza viruses (Xue et al., 2018). In contrast, the impact of within-host virus diversity and infection severity has been rarely reported among coronaviruses. Therefore, we assessed within-host diversity of SARS-CoV-2 viruses in COVID-19 patients with variable disease severities.

In accordance with other studies, analysis of viral sequences at the consensus level revealed high similarities of all viruses regardless of disease severity (Shen et al., 2020). Nonetheless, in-depth analysis revealed the presence of sub-consensus variants in both mild and severe cases but at significantly higher numbers among severe cases. The existence of viral quasispecies has been reported, although not frequently, in COVID-19 patients (Capobianchi et al., 2020; Ceraolo and Giorgi, 2020; Shen et al., 2020; van Dorp et al., 2020). One of the earliest studies that were carried in Wuhan during December 2019 also reported the presence of within-host variants (range from 0 to 51 variants per patient) in hospitalized COVID-19 patients (Shen et al., 2020). Similarly, analysis of the first two cases from Lombardy, Italy, also revealed the presence of virus quasispecies; however, with limited variation compared to our study (Capobianchi et al., 2020). The lower number of reported within-host variants in the latter study is most probably related to the small number of samples, the different sequencing protocols, and the low coverage of sequencing reads.

Despite the diversity in SARS-CoV-2 genomes at the sub-consensus level, we were not able to identify genomic variability hotspots at the sub-consensus levels. Overall, very few within-host variants were detected in more than two patients, which may suggest a narrow transmission bottleneck or purifying selection of variants. The latter possibility is further supported by our results, which showed a significantly high number of non-synonymous variants at the sub-consensus level, none of which were seen at the consensus level. Similar findings were also reported in other SARS-CoV-2 studies (Sigal et al., 2018; Capobianchi et al., 2020; Shen et al., 2020). Still, other research groups were able to find variability hotspots in SARS-CoV-2 genomes (Ceraolo and Giorgi, 2020; van Dorp et al., 2020). Considering SARS-CoV-2 consensus sequences, we were able to identify three major mutations hotspots in both mild and severe cases: 11,083 and 14,805 in ORF1ab and 26,144 in ORF3a. Of these, 14,085, in the RNA-dependent RNA polymerase (RdRp) coding region, was not found to be a variation hotspot in SARS-CoV-2 sequences deposited in GISAID (https://nextstrain.org/ncov/global). However, this is not the first study to report its appearance (Korber et al., 2020; Pachetti et al., 2020). Additionally, a study that has been carried out by Eskier et al. (2020) indicated that C14408T mutation, in RdRp, can increase the mutation rate in SARS-CoV-2 viruses (Eskier et al., 2020). Less prevalent hotspots were also reported among patients of both groups (Figure 4). Of those, variants 14,408 (RdRp), 23,403 (S), and 28,881 (N) were found to be global mutations hotspots according to GISAID; however, they were predominantly observed in Europe (Pachetti et al., 2020; Yao et al., 2020).

The spike (S) glycoprotein mediates virus entry into host cells and is thought to be the major determinant of host tropism (Fung and Liu, 2019). Further, the S protein is the main target of neutralizing antibodies and hence is under constant pressure to mutate (Delmas et al., 1990; Zhang et al., 2004; Yu et al., 2020). Therefore, we expected to find more mutations in the S gene compared to other genes; instead, the distribution of variants was even across the whole genome. Moreover, none of the S variants—neither at consensus nor at sub-consensus level—was located in the receptor-binding domain (RBD) or in the epitope residues of recently identified neutralizing antibodies, CR3022 and 47RD11 (Wang et al., 2020; Yuan et al., 2020). This might indicate a potential virus adaption to its new host. It also shows that the virus can be effectively be targeted using these antibodies.

The lack of significant variations in virus genomes at the consensus level has provided researchers with the reason to believe that disease severity is mainly determined by host factors such as age, comorbidities, and the uncontrolled immune response (Guan et al., 2020b; Spychalski et al., 2020). So far, age is considered the main determinant of disease severity (Guan et al., 2020b). Globally, the fatality rate was found to be 1.4% in infected patients under the age of 60, 4.5% in patients above 60, and reaching up to 13% in patients above the age of 80 (Spychalski et al., 2020; Verity et al., 2020). In our study, 34% of severe cases belonged to patients above the age of 60, while none of the mild cases was above the age of 54. Interestingly, assessing within-host virus diversity among different age groups has also revealed higher within-host diversity among patients above the age of 60 and among patients aged between 43 and 49 years. This could be related to the relatively weak immune systems, especially among elderlies, which may not be able to efficiently clear the infection, allowing the virus to replicate to high diversity. These observations suggest that higher within-host diversity may further contribute to severe manifestations commonly seen among elderlies. Further, in accordance to other studies, the number of patients with at least a single comorbidity was higher among severe cases (62%) compared to mild cases (29%), with hypertension being the most common comorbidity (Grasselli et al., 2020; Guan et al., 2020a,b; Vardavas and Nikitara, 2020). Unlike the age factor, though, there was no significant association between comorbidities and within-host diversity. Instead, severe cases exhibited higher within-host diversity—compared to mild cases—regardless of the number of associated comorbidities. The uncontrolled hyperactivation of the immune response has been also shown to contribute to severe manifestations seen in SARS-Cov-2 infection (Wu et al., 2009; Feldmann et al., 2020; Mehta et al., 2020). The overstimulation of the immune response—mainly cytokines—has also been documented in SARS and MERS patients (Tisoncik et al., 2012; van den Brand et al., 2015). It is worth noting that the presence of the genetically diverse quasispecies is also expected to result in the production of a wide range of antibodies to encounter virus diversity. Here, we observed significantly higher levels (*p*-value < 0.0001) of within-host diversity among severe cases compared to mild cases. The presence of these variants, even at frequencies as low as 10%, might trigger immune response to produce non-specific cytokines or variant-specific antibodies, resulting in enhanced immune response and severe clinical outcomes (Huang et al., 2011; Gong et al., 2020). However, further investigations to confirm the presence of these quasispecies-specific antibodies is required to support this hypothesis. Also, the high diversity of the virus could potentially increase the fitness of the viral population, making it hard to eradicate (Domingo et al., 2012; Berngruber et al., 2013). So far, it is unclear whether these within-host variants occurred as a result of immune pressure, or they triggered a more aggressive immune response, which would result in different conclusions. Therefore, additional studies are needed to explore how this may influence the immune response toward the virus and whether there is a selection acting on different variants in infected hosts or during the transmission.

In conclusion, exploring within-host diversity of this newly emerged virus, SARS-CoV-2, has revealed significant differences between mild and severe cases, particularly among older patients. Therefore, further investigation on the within-host diversity role in disease severity is of significance at this stage of the pandemic and should be considered in future studies. Further studies should be conducted using a higher number of samples collected from patients with variable disease severity and at different age groups to confirm findings of this study on a large scale. It is also crucial to compare viral quasispecies in patients of different age groups as well as from different sample types. This would also help us better understand disease severity and transmission patterns.

## Data Availability Statement

All sequencing fastq files are available in the National Center for Biotechnology Information (NCBI) under accession number PRJNA639864 (https://www.ncbi.nlm.nih.gov/bioproject/, PRJNA639864).

## Ethics Statement

The studies involving human participants were reviewed and approved by QU and HMC ethical boards as mentioned in the manuscript. Written informed consent for participation was not required for this study in accordance with the national legislation and the institutional requirements.

## Author Contributions

HY and HA designed the concept. HA ran the sequencing, analyzed the data, and wrote the first manuscript draft. FB collected the demographic and clinical data. IE participated in the sequencing experiments. AA provided the funding. AA-K, MA, and PC helped in sample collection and other logistics. All authors contributed to the article and approved the submitted version.

## Conflict of Interest

The authors declare that the research was conducted in the absence of any commercial or financial relationships that could be construed as a potential conflict of interest.
